# Integrating stress responses and secondary metabolism: the regulatory functions of histone deacetylases in medicinal plants

**DOI:** 10.3389/fpls.2026.1845669

**Published:** 2026-05-28

**Authors:** Yingdie Yang, Duoteng Tang, Rongxian Zhang, Mingchun Liu, Tu Feng

**Affiliations:** 1 School of Ecological Engineering, Guizhou University of Engineering Science, Bijie, China; 2 Key Laboratory of Bio-Resource and Eco-Environment of Ministry of Education, College of Life Sciences, Sichuan University, Chengdu, China; 3 College of Life and Health Science, Kaili University, Kaili, Guizhou, China

**Keywords:** abiotic stress, epigenetic regulation, histone deacetylase, medicinal plant, secondary metabolism, stress signaling

## Abstract

Medicinal plants are an important source of pharmacologically active secondary metabolites. However, their cultivation is often constrained by environmental stresses such as extreme temperatures, drought, salinity and ultraviolet radiation. These stresses reshape the biosynthesis of secondary metabolites including alkaloids, phenolic compounds and terpenoids through coordinated signaling, transcriptional reprogramming and chromatin remodeling. Epigenetic regulation provides a reversible mechanism by which plants translate environmental cues into stable but adjustable gene-expression states. Among epigenetic regulators, histone deacetylases have emerged as important modulators of stress responses and specialized metabolism. This review summarizes current knowledge of histone deacetylase families in plants, with emphasis on their roles in stress signaling and secondary metabolite biosynthesis in medicinal plants. We discuss evidence from phenylpropanoid, terpenoid and alkaloid-related pathways, and distinguish transcriptional mechanisms from post-translational regulation of metabolic enzymes. We further propose an integrative framework in which histone deacetylases function as regulatory nodes that connect hormone signaling, stress-responsive transcription factors, chromatin accessibility and metabolic flux. This framework provides insight into plant adaptation and suggests potential strategies for improving the yield and stability of bioactive compounds in medicinal plants.

## Background

Medicinal plants have long been used as a basic source of traditional healthcare systems and continue to play a vital role in the discovery and development of modern therapeutics (Dar et al., 2017). Historical records from ancient civilizations, including China, India, Egypt and Greece, all showed the extensive use of botanical resources for the treatment and prevention of diseases (Hamilton, 2004). These plants are characterized by their production of diverse bioactive secondary metabolites, including alkaloids, flavonoids, terpenoids, glycosides and phenolic compounds, many of which exhibit significant pharmacological activities (Hussein and El-Anssary, 2019). The transition of medicinal plants from traditional medicines to modern pharmacology began in the 19th century with the isolation of active compounds such as morphine from opium poppy (*Papaver somniferum*), quinine from *Cinchona species*, and salicin from *Salix* species (Chaachouay and Zidane, 2024). These discoveries marked the beginning of evidence-based medicine and drove the development of pharmacology as a scientific discipline. Today, plant-derived compounds remain essential in drug development, and traditional medicine continues to serve as a primary healthcare resource for a significant portion of the global population, particularly in developing countries (Rout et al., 2009).

Secondary metabolites (SMs) are bioactive compounds synthesized by medicinal plants. Extensive research have indicated the therapeutic activities of SMs in animal and human models of various diseases (Velu et al., 2018). In addition to that, SMs also serve as significant components in the plant’s response to biotic and abiotic stresses, contributing to stress adaptation through antimicrobial, antioxidant and protective activities (Ibragić et al., 2025). However, their biosynthesis is highly sensitive to environmental factors such as drought, salinity, and temperature fluctuations, which often limit the productivity and quality of medicinal plants. Under stress, plants therefore need to coordinate two processes that may compete for resources: maintaining growth and redirecting metabolic flux toward protective compounds. Understanding how this coordination is achieved is essential for improving the quality and stability of medicinal plant production.

Epigenetic regulation provides an important layer of this coordination. Epigenetic modifications refer to heritable or reversible changes in gene activity that occur without altering the underlying DNA sequence. In plants, major epigenetic mechanisms include DNA methylation, histone methylation, histone acetylation and deacetylation, chromatin remodeling and small-RNA-mediated regulation (Singh et al., 2023; Ibragić et al., 2025). These mechanisms influence chromatin accessibility and thereby determine whether stress-responsive and metabolic genes are transcriptionally active or repressed.

Histone acetylation is especially relevant to rapid stress responses. Histone acetyltransferases add acetyl groups to lysine residues on histones, generally loosening chromatin and promoting transcription, whereas histone deacetylases (HDACs) remove acetyl groups and often restore a compact chromatin state (Ma et al., 2013; Liu et al., 2014; Cui et al., 2023). HDACs, in particular, have emerged as central regulators that integrate environmental signals with transcriptional and metabolic responses (Chen et al., 2024; Ibragić et al., 2025). Since secondary metabolite genes are frequently activated under stress and repressed after stress relief, histone acetylation dynamics provide a plausible mechanism for linking environmental signals with metabolic reprogramming. However, the mechanistic links between HDAC-mediated epigenetic regulation and secondary metabolite biosynthesis remain incompletely understood.

In this review, we focus on HDACs as regulatory nodes connecting stress signaling, chromatin state and secondary metabolism in medicinal plants. We synthesize current knowledge on HDAC function in medicinal plants and propose an integrative regulatory model linking stress signaling, epigenetic modulation and secondary metabolism. This framework provides new insights into plant adaptive strategies and highlights potential targets for improving metabolite production through biotechnological approaches.

## Epigenetic regulation of stress responses in medicinal plants

Plants respond to stress through complex regulatory networks that involve extensive transcriptional reprogramming. Epigenetic mechanisms, including DNA methylation, histone modification, and small RNA-mediated regulation, play essential roles in controlling these responses without altering the underlying DNA sequence (Singh et al., 2023; Ibragić et al., 2025).

DNA methylation is generally associated with transcriptional repression, whereas histone acetylation is usually associated with open chromatin and active transcription (Singh et al., 2023). Histone deacetylation can repress gene expression by reducing histone acetylation, promoting nucleosome compaction and limiting transcription-factor access to target promoters. These reactions are reversible, allowing plants to rapidly activate stress-protective genes and then return to basal expression after the stress relief.

Plant HDACs are classified into three major families: reduced potassium dependency 3/histone deacetylase 1 (RPD3/HDA1), silent information regulator 2 (SIR2) and the plant-specific histone deacetylase 2 (HD2) family, each distinguished by their structural features, catalytic mechanisms and functional roles in plant responses to environmental stress (Ma et al., 2013; Liu et al., 2014). The RPD3/HDA1 family is the largest group and contains a conserved histone deacetylase catalytic domain. Its members are usually zinc-dependent enzymes and are often subdivided into class I, class II and class IV groups based on sequence similarity (Ma et al., 2013; Liu et al., 2014) These proteins commonly function in transcriptional repression by forming complexes with transcription factors or co-repressors. The SIR2 family contains a conserved sirtuin domain and requires nicotinamide adenine dinucleotide as a cofactor, linking deacetylase activity with cellular energy status and metabolic regulation (Ma et al., 2013; Liu et al., 2014). The plant-specific HD2 family is structurally distinct from the other two families and is characterized by an amino-terminal pentapeptide motif and an acidic region. Many members contain a conserved NPL domain and may also possess zinc-finger-like regions that facilitate chromatin or protein interactions (Ma et al., 2013; Liu et al., 2014; Yang et al., 2021). These structural differences suggest that the three families can respond to different upstream signals and regulate different downstream metabolic pathways.

Studies in model plants provide mechanistic examples that clarify how histone deacetylases act during stress. In Arabidopsis thaliana, histone deacetylases such as HDA6, HDA19, HDA15, and HD2A/HD2B participate in abscisic-acid, salt, and drought responses by modulating histone acetylation at stress-responsive loci (Chen et al., 2010; Lee and Seo, 2019; Zhao et al., 2022). For example, the MYB96-HDA15 complex represses RHO OF PLANTS genes by removing acetyl groups from histone H3 and H4, thereby strengthening abscisic-acid responses and drought tolerance (Lee and Seo, 2019). In rice (*Oryza sativa*), HDA710 regulates abscisic-acid- and salt-responsive genes by altering histone H4 acetylation at promoters, while OsWR2 recruits HDA704 to deacetylate histone H4 lysine 8 at the OsABI5 promoter during drought stress (Ullah et al., 2021; Guo et al., 2023). These examples show that histone deacetylases usually do not act alone; rather, they are recruited by stress-responsive transcription factors and co-regulators to specific genomic regions.

In medicinal plants, evidence is accumulating but remains uneven across species. In Shihu (*Dendrobium officinale*), 14 HDAC genes have been identified and their expressions (*DoHDA3*, *DoHDA8*, and *DoHDT4*) respond distinctly under abiotic stresses (*e.g.* drought and salt) and phytohormone treatments such as abscisic acid (ABA) and methyl jasmonate (MeJA) (Zhang et al., 2020). Similarly, in Danshen (*Salvia miltiorrhiza*), 16 *SmHDAC* genes were identified, with family members showing divergent responses to different abiotic stresses (drought and salt stress) and stress hormone (ABA and MeJA) treatments (Chen et al., 2024). In balloon flower (*Platycodon grandiflorus*), histone deacetylase family members respond to waterlogging stress, indicating their potential contribution to stress adaptation in root tissues (Ahn et al., 2023b). In Tartary buckwheat (*Fagopyrum tataricum*), *FtHDA6* is implicated in response to low temperature stress (Hou et al., 2022). Together, these studies indicate that medicinal plants differ in histone deacetylase copy number, family composition, and stress inducibility. As a result, comparative analysis is needed to generalize mechanisms across species.

A comparison among representative medicinal plants suggests three regulatory patterns. First, the RPD3/HDA1 family tends to be the most expanded and stress-responsive group, consistent with its broad role in transcriptional repression. Second, SIR2 genes may link stress responses with carbon and energy metabolism, which is particularly relevant for secondary metabolite biosynthesis. Third, plant-specific HD2 genes often respond to water-related stresses and may provide plant-lineage-specific mechanisms for chromatin adaptation (Luo et al., 2017; Bajpai et al., 2024). Danshen (*Salvia miltiorrhiza*) is a useful example because it produces clinically important phenolic acids and diterpenoid quinones, with its histone deacetylase genes responding to both abiotic stress and stress hormones (Chen et al., 2024). This makes it an appropriate system for testing whether stress-induced histone deacetylase changes directly modulate tanshinone and phenolic-acid biosynthetic genes.

**Figure 1:**
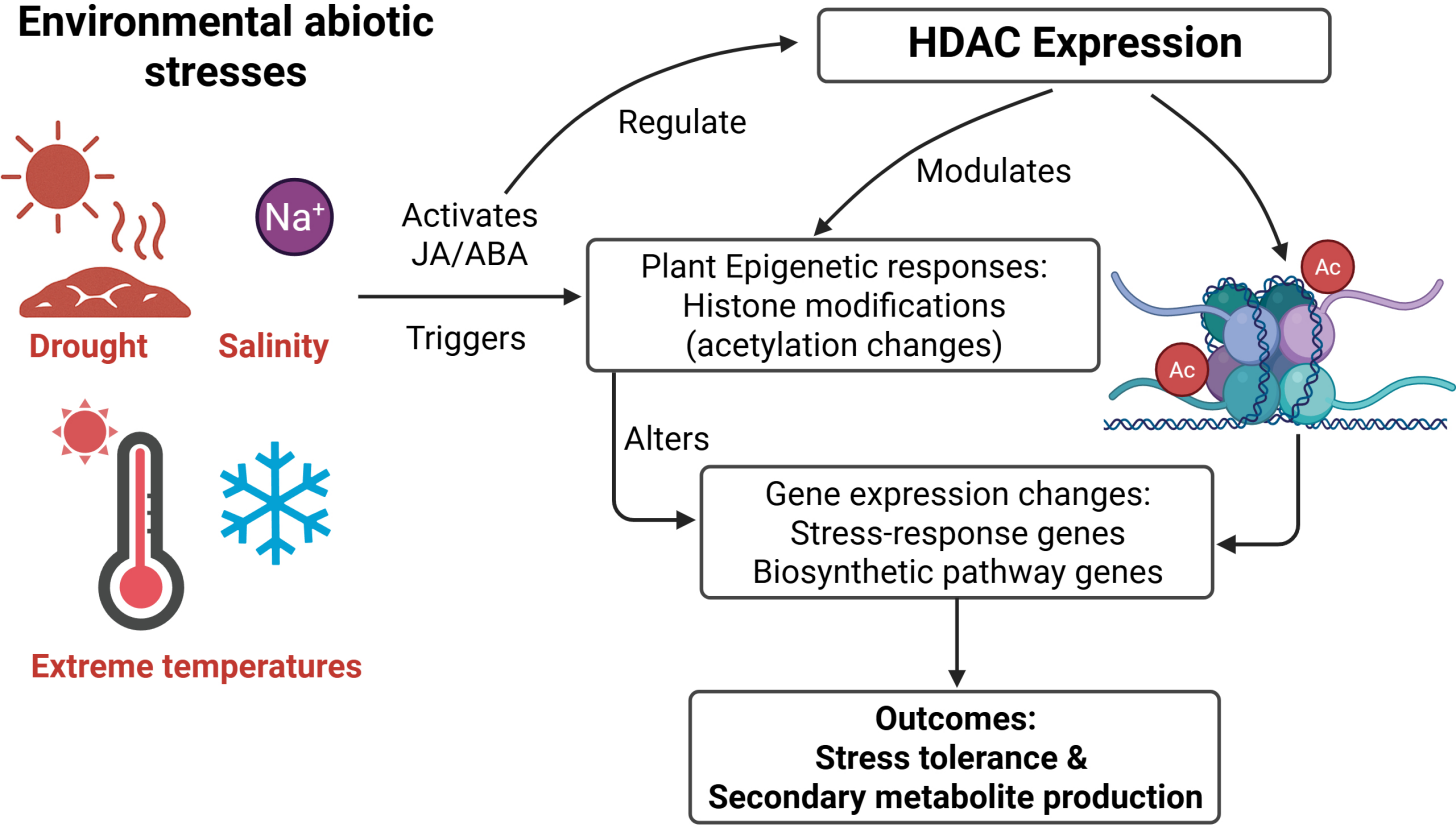
The principal function of plant HDAC in response to environmental stresses. Abiotic stresses activate hormone and reactive oxygen species signaling. These signals alter histone deacetylase expression, recruitment, or activity, thereby changing histone acetylation at stress-responsive genes and modulating adaptive gene expression.

Mechanistically, environmental stimuli such as drought, salinity and extreme temperature will activate stress signals such as ABA, MeJA and reactive oxygen species, which influencing histone deacetylase expression, protein recruitment and enzyme activity (**Figure 1**). Stress-responsive transcription factors from the MYB, WRKY, bZIP, AP2/ERF, NAC and basic helix-loop-helix families may recruit histone deacetylases to target promoters, where the removal of histone acetylation marks can repress negative regulators or reset previously activated genes (Hussein and El-Anssary, 2019; Lee and Seo, 2019; Jan et al., 2021; Guo et al., 2023). Therefore, histone deacetylases should be viewed not only as expression markers but also as enzymes that define chromatin states at stress-response and metabolic loci.

## Secondary metabolites in medicinal plants

Secondary metabolites are structurally diverse compounds that play essential roles in plant adaptation and ecological interactions (Tsipinana et al., 2023; Ibragić et al., 2025). Unlike primary metabolites (sugars, amino acids and lipids), which are required for basic cellular functions, secondary metabolites are often synthesized in response to environmental challenges such as herbivory, pathogen attack and abiotic stress. Under unfavorable conditions, plants tend to synthesize more resources into secondary metabolism as a defense strategy (Tsipinana et al., 2023). These compounds can deter herbivores and microbes, protect against UV radiation, or act as signals to attract pollinators and seed dispersers. For example, many floral pigments (e.g. anthocyanins) and fragrances (volatile terpenoids) are secondary metabolites that aid in pollinator attraction, while others (like bitter alkaloids or phenolics) ward off pests (Salam et al., 2023).

Plant secondary metabolites are extremely diverse, but they can be grouped into several broad classes based on their structures and biosynthetic origins (Tsipinana et al., 2023). The principal categories include alkaloids, phenolics and terpenoids. Each class arises *via* specific biosynthetic pathways and serves particular functions. Usually, plants utilize a few core pathways to generate these diverse structures: the shikimate pathway, the acetate–polyketide pathway, and the mevalonate (isoprenoid) pathway (including the related MEP pathway) (Tsipinana et al., 2023). The shikimate pathway provides aromatic precursors (e.g. aromatic amino acids) for many phenolics and some alkaloids; the polyketide pathway (using acetyl-CoA/malonyl-CoA building blocks) yields phenolics, flavonoids and quinones; the mevalonate (MVA) and methylerythritol phosphate (MEP) pathways produce the isopentenyl diphosphate units for terpenoids. Below, we discuss the major classes in detail.

### Alkaloids

Alkaloids represent one of the largest classes of plant secondary metabolites, with over 20,000 distinct alkaloids identified from plants (Ziegler and Facchini, 2008; Tsipinana et al., 2023). Alkaloids are nitrogen-containing secondary metabolites typically derived from amino acid precursors (*e.g.* tryptophan, tyrosine and ornithine) through specialized branch pathways (Bhambhani et al., 2021). In addition to their well-known pharmacological usages, such as analgesic (morphine from opium poppy, *Papaver somniferum*), anti-cancer (vinblastine from *Catharanthus roseus*) and anti-malarial (quinine from *Cinchona* bark) effects, alkaloids are also important for plant defense to environmental stresses (Achan et al., 2011; Labanca et al., 2018; Taher et al., 2019; Bhambhani et al., 2021).

**Figure 2:**
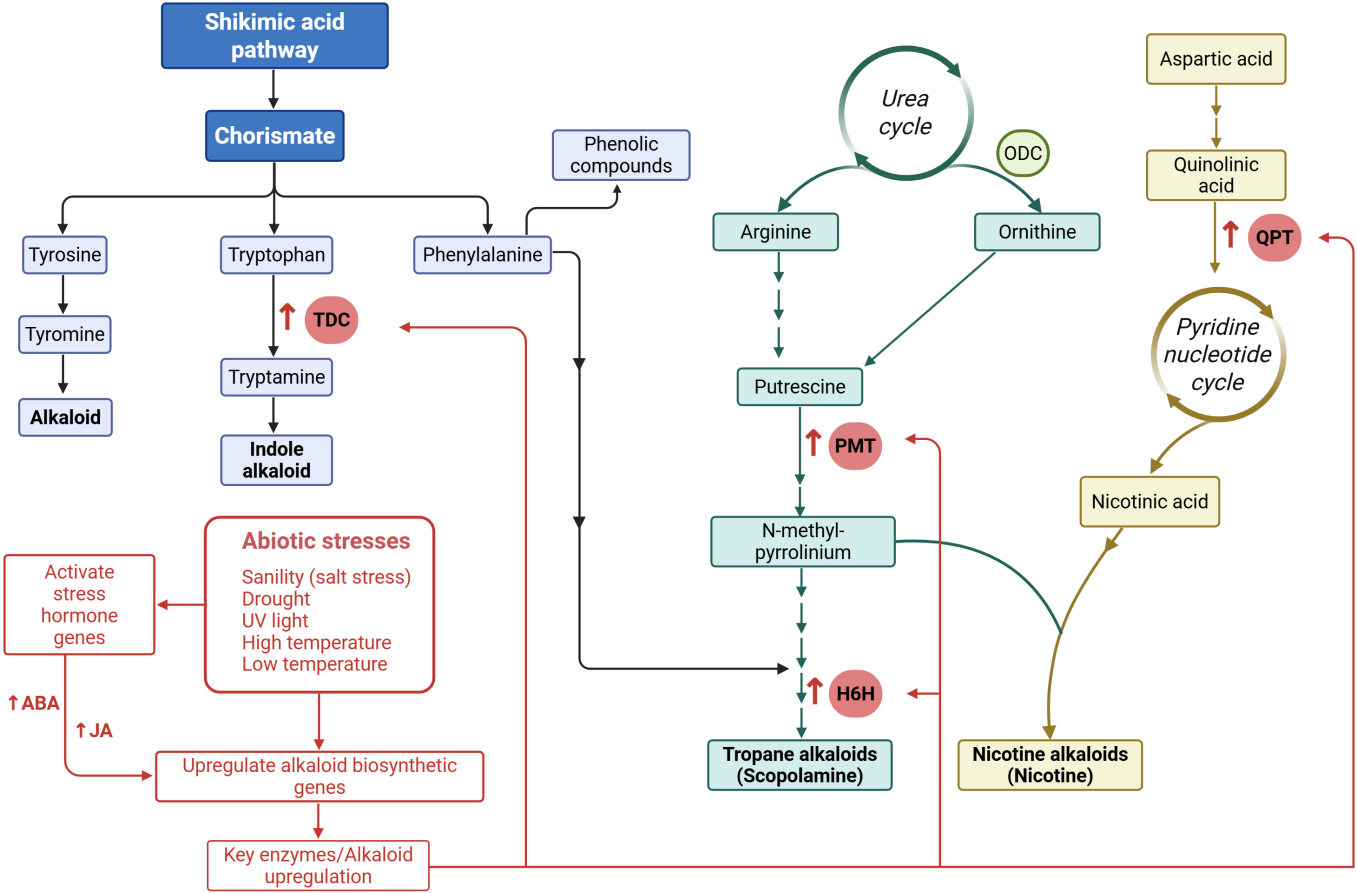
Plant alkaloid biosynthetic pathways and expression modulation under stresses. Alkaloids are derived mainly from shikimate-, ornithine/arginine-, and pyridine-nucleotide-related pathways. Abiotic stress activates stress hormones ABA and MeJA, then mediate the expression of key enzymes including tryptophan decarboxylase (TDC), putrescine N-methyltransferase (PMT), hyoscyamine 6β-hydroxylase (H6H), and quinolinate phosphoribosyltransferase (QPT).

In normal conditions, plants synthesize alkaloids through several multi-step enzymatic routes: the shikimic acid pathway, where tryptophan decarboxylase (TDC) converts tryptophan to tryptamine, initiating indole alkaloid biosynthesis (**Figure 2**). The ornithine decarboxylase (ODC) and putrescine N-methyltransferase (PMT) initiate tropane and pyridine alkaloid formation from ornithine/arginine (Ryan et al., 2015; Reshi et al., 2023) and the quinolinate phosphoribosyltransferase (QPT) is responsible for nicotine alkaloids biosynthesis. These pathways often involve decarboxylation, methylation, and oxidation steps catalyzed by key enzymes such as PMT and QPT for nicotine, hyoscyamine 6β-hydroxylase (H6H) for converting hyoscyamine to scopolamine in the tropane alkaloid pathway (Dehghan et al., 2013; Ryan et al., 2015). Under abiotic stresses (drought, salinity and extreme temperature), plants usually upregulate alkaloid biosynthetic genes and enzymes, leading to higher alkaloid accumulation as a stress responsive mechanism (Gong et al., 2024) (**Figure 2**). Mechanistically, abiotic stresses will mediate the expression of stress hormones like JA and ABA, then these hormones will mediate the induction of alkaloid pathway genes (and key enzymes) to upregulate alkaloid biosynthesis. For example, in *Chelidonium majus*, the alkaloid dihydrocoptisine concentration rose significantly under drought and salinity stress (Yahyazadeh et al., 2018; Gong et al., 2024). Moreover, it was found that salt stress can enhance H6H expression then up-regulate tropane alkaloid production. Moreover, UV stress can induce TDC activity to promote indole alkaloid synthesis (Martens et al., 2010; Reshi et al., 2023). These changes result in elevated biosynthetic flux through alkaloid pathways during stress, as indicated by increased transcript levels of enzymes like PMT, QPT and H6H in stressed roots and tissues (Ryan et al., 2015; Gong et al., 2024) (**Figure 2**). The accumulated alkaloids under stress not only deter herbivores but may also help plants alleviate oxidative damage and other stress effects (Gong et al., 2024).

### Phenolic compounds (phenolics and polyphenols)

Phenolics constitute another big group of plant secondary metabolites, characterized by one or more aromatic rings bearing hydroxyl groups. Phenolics can range from simple phenolic acids to complex polyphenols. Major subclasses include flavonoids, stilbenes, coumarins, tannins (polymeric phenolics), lignins (structural polymers in wood) and anthocyanins (pigments). Phenolics are known for their health benefits when consumed in the human diet. For instance, flavonoids, such as quercetin and catechins, have shown anti-inflammatory, anti-viral, and cardioprotective effects (Al-Khayri et al., 2022). In addition, phenolic compounds also have numerous roles within plants. For instance, they contribute to pigmentation (e.g. red, purple, and blue colors of flowers and fruits due to anthocyanins) which aids in attracting pollinators and seed dispersers (Babenko et al., 2019) and also contribute to the defense mechanism of plants to environmental stresses (Sharma et al., 2019).

**Figure 3:**
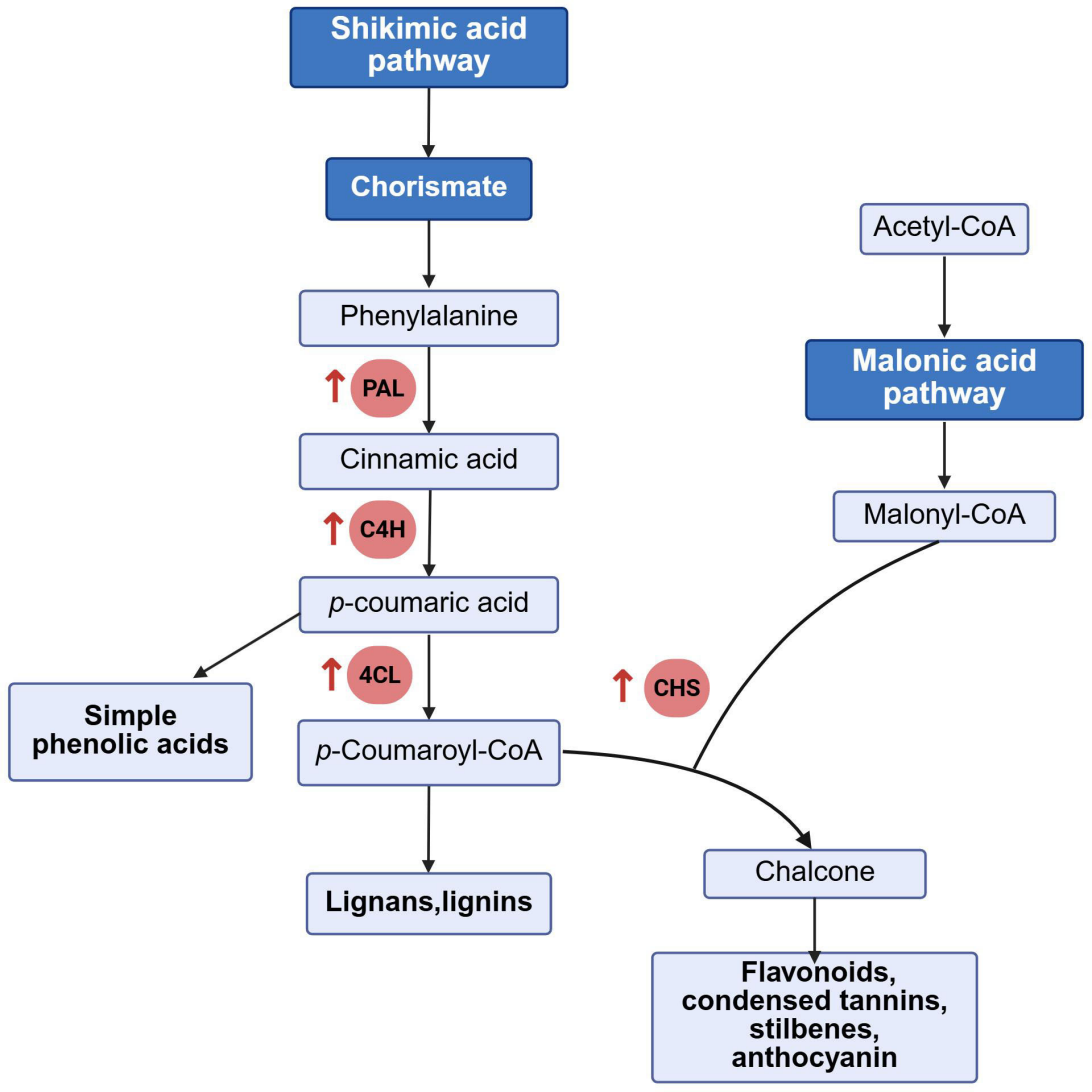
Plant phenolic compounds biosynthetic pathways and expression modulation under stresses. Phenolic compounds are produced through shikimate/phenylpropanoid and malonyl-CoA-derived branches. Stress commonly induces expression of key enzymes such as PAL (phenylalanine ammonia-lyase), C4H (cinnamate 4-hydroxylase), 4CL (4-coumarate CoA ligase) and CHS (chalcone synthase).

Phenolic compounds are mainly synthesized *via* the shikimate/phenylpropanoid pathway, starting from the aromatic amino acid phenylalanine (Saltveit, 2017). Phenylalanine is deaminated by phenylalanine ammonia-lyase (PAL) to form cinnamic acid, which is then hydroxylated by cinnamate 4-hydroxylase (C4H) and activated to 4-coumaroyl-CoA by 4-coumarate CoA ligase (4CL) (Liu et al., 2023) (**Figure 3**). These early steps lead into two branch routes: one branch produces flavonoids *via* chalcone synthase (CHS) and downstream enzymes (Sharma et al., 2019; Liu et al., 2023). Another branch yields monolignols for lignin *via* enzymes like cinnamoyl-CoA reductase (CCR) and cinnamyl alcohol dehydrogenase (CAD) (Liu et al., 2023). Under abiotic stress, plants often show transcriptional upregulation of PAL, C4H, 4CL, CHS, CHI and related enzymes, leading to higher phenolic content as a protective response (Sharma et al., 2019). For example, it was found that drought stress in potato tubers upregulated genes encoding PAL, HCT, C3H, CHS, CHI, F3H, DFR and MYB regulator AN1 (André et al., 2009; Kumar et al., 2023). Salinity stress similarly triggers phenolic production. In tobacco, a specific CHS isoform (NtCHS1) was upregulated under salt, resulting in increased flavonoid levels (Bistgani et al., 2019). These induced phenolics may function as antioxidants, osmo-protectants, and structural barriers to mitigate stress damage.

### Terpenoids (isoprenoids)

Terpenoids are a huge class of secondary metabolites derived from five-carbon isoprene units. Medicinally, terpenoids have given rise to some of the most important drugs. For example, paclitaxel (Taxol) is a complex diterpene isolated from the Pacific yew tree (*Taxus brevifolia*), which is an effective anti-cancer drug (Suffness and Wall, 2021). Artemisinin (*Artemisia annua*), a sesquiterpene lactone, used in Chinese medicine, is a powerful antimalarial drug and has also shown anticancer properties (Banožić et al., 2023). Ecologically, terpenoids have diverse roles. Many terpenes act as essential oils that either deter herbivores or attract pollinating insects through their distinctive aromas, such as menthol, limonene, and pinene (Metcalf and Kogan, 1987). Other terpenoids, such as carotenoids, contribute to floral and fruit pigmentation, producing yellow to orange colors that serve as visual attractants, while also functioning as potent antioxidants (Tholl, 2015; Li et al., 2023). In addition, some terpenoids also function as hormones within the plant (e.g. gibberellins and ABA) or as signaling molecules in plant defense (e.g. phytoalexins like capsidiol).

**Figure 4:**
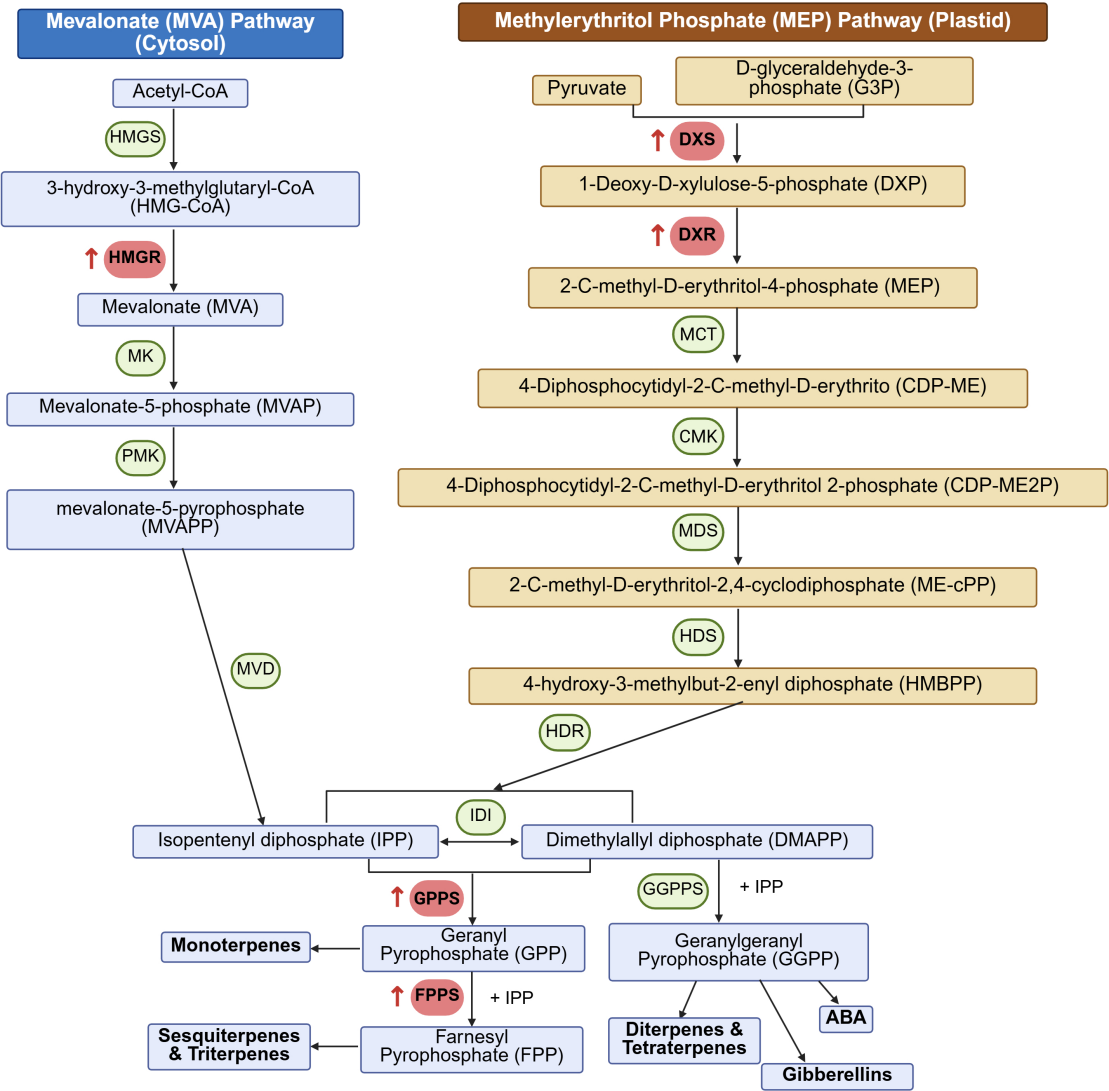
Plant terpenoids biosynthetic pathways and expression modulation under stresses. The mevalonate and methylerythritol phosphate pathways supply isopentenyl diphosphate and dimethylallyl diphosphate for terpenoid biosynthesis. Stress can activate key enzymes such as 3-hydroxy-3-methylglutaryl-CoA (HMGR), 1-deoxy-D-xylulose-5-phosphate synthase (DXS), 1-deoxy-D-xylulose-5-phosphate reductoisomerase (DXR), geranyl pyrophosphate synthase (GPPS) and farnesyl pyrophosphate synthase (FPPS).

Generally, the biosynthesis of terpenoids in plants is through two pathways, the cytosolic mevalonic acid (MVA) and plastidial 2-C-methyl-D-erythritol-4-phosphate (MEP) pathways (also named as methylerythritol phosphate pathway). Under normal conditions, the MVA pathway starts from acetyl-CoA to 3-hydroxy-3-methylglutaryl-CoA (HMG-CoA), which is then reduced by 3-hydroxy-3-methylglutaryl-CoA reductase (HMGR) to mevalonate. Mevalonate is phosphorylated and decarboxylated to yield IPP (isopentenyl pyrophosphate), which interconverts with DMAPP (dimethylallyl pyrophosphate). IPP and DMAPP are important precursors for the downstream biosynthesis of various terpenes (Vranová et al., 2013; Tholl, 2015) (**Figure 4**). Meanwhile, the plastid MEP pathway (non-mevalonate pathway) starts from combining pyruvate and glyceraldehyde-3-phosphate (G3P) to form 1-Deoxy-D-xylulose-5-phosphate (DXP) *via* DXP synthase (DXS). The DXP is converted to MEP (2-C-methyl-D-erythritol-4-phosphate) by DXR (1-deoxy-D-xylulose-5-phosphate reductoisomerase) and eventually IPP/DMAPP in plastids (Vranová et al., 2013; Tholl, 2015). The MEP pathway predominantly supplies precursors for monoterpenes, diterpenes, carotenoids, and isoprene in plastids, while the MVA pathway supplies sesquiterpenes, sterols and triterpenoids in cytosol (Vranová et al., 2013; Tholl, 2015; Reshi et al., 2023).

Under abiotic stress, terpenoid biosynthesis is often upregulated, similar to alkaloids and phenolics. Key enzymes involved in both MEP and MVA pathways such as HMGR, DXS, DXR and downstream GPPS as well as FPPS, are often upregulated and lead to increased expression of protective terpenoid such as sterols (maintain membrane integrity and fluidity) or hormonal terpenoids to regulate stress response (Simpson et al., 2018; Ali et al., 2021; Huang et al., 2021) (**Figure 4**). For example, heat stress resulted in increased isoprene emission through upregulation of the MEP pathway, which is believed to stabilize membranes and counteract heat damage (Singsaas, 2000; Zuo et al., 2025). Drought stress and salinity can also influence terpenoid pathways. It was found drought-exposed plants showed more than 20% increase in phytol (diterpenoid) levels and transcriptomic study in slash pine identified transcription factors (MYB, AP2/ERF, WRKY, bHLH) and enzymes (HMGCR, MVK, GPS, DXP, GGPS, TPS) involved in terpenoid biosynthesis under such condition (Reshi et al., 2023; Zhang et al., 2023). Moreover, salt stress can stimulate production of specific isoprenoids that function in osmoprotection or stress signaling (e.g. abscisic acid (ABA), a terpenoid hormone, rises under salinity).

These examples demonstrate that stress-induced secondary metabolism is controlled not only by pathway enzymes and transcription factors but also by chromatin state. Epigenetic regulation provides the reversible switch that determines whether alkaloid, phenylpropanoid and terpenoid biosynthetic genes remain basal, become stress-inducible, or return to repression after stress relief. Therefore, the next key question is how histone deacetylases and related chromatin regulators interface with the metabolic genes discussed above.

## Histone deacetylase mediated epigenetic regulation of secondary metabolite pathway

Plants use multiple epigenetic mechanisms to regulate secondary metabolism, including DNA methylation, histone modification, chromatin remodeling, and small-RNA-mediated regulation. Histone deacetylation is particularly important because it directly changes chromatin accessibility and can also influence the acetylation status of non-histone proteins (Ma et al., 2013; Dar et al., 2017). Therefore, HDAC may regulate secondary metabolism through two interconnected mechanisms: transcriptional control of biosynthetic genes and post-translational control of metabolic enzymes.

At the transcriptional level, HDACs are generally recruited to promoters by sequence-specific transcription factors or co-repressors rather than by direct DNA recognition. Their catalytic activity reduces histone acetylation marks such as H3K9ac, H3K14ac, H3K27ac, and H4K8ac, promoting chromatin compaction and repressing transcription. Stress hormones or HDAC inhibitors can relieve this repression, resulting in higher histone acetylation, improved transcription-factor access and activation of biosynthetic genes (Zheng et al., 2016; Hu et al., 2019; Bajpai et al., 2024). At the post-translational level, some histone deacetylases deacetylate metabolic enzymes directly, thereby altering enzyme stability, activity, or substrate flux (Yang et al., 2021; Alimirzaee et al., 2024). These two levels can be synergistic when both gene expression and enzyme activity are enhanced, but they can also appear antagonistic when chromatin-level repression and enzyme-level activation occur in the same pathway.

### Phenylpropanoid pathways and HDAC modulation

HDACs often act as repressors of phenylpropanoid biosynthetic genes under non-stressed conditions and relief of this repression can increase such metabolite accumulation. For example, in balloon flower (*Platycodon grandiflorus*), treatment of root cultures with sodium butyrate (NaB), a broad HDAC inhibitor, significantly up-regulated many genes in the phenylpropanoid biosynthesis pathway (Ahn et al., 2023a). This transcriptional activation was accompanied by increased accumulation of downstream phenolics, including dihydroquercetin and gallic acid, compared with untreated controls (Ahn et al., 2023a). The coordinated increase in pathway-gene expression and metabolite levels suggests that histone deacetylase inhibition de-represses phenylpropanoid biosynthesis. Similarly, in licorice (*Glycyrrhiza inflata*), the sirtuin-type histone deacetylase GiSRT2 acts as a negative regulator of flavonoid biosynthesis. Nicotinamide (HDAC inhibitor) treatment or GiSRT2 knockdown increased licochalcone A (a retrochalcone flavonoid) accumulation and de-repressed pathway genes such as GiLMT1 (Zeng et al., 2023; Zeng et al., 2025). In bamboo suspension cells, adding HDAC inhibitors resulted in high production of phenolic compounds that are usually nearly undetectable in those cells, further supporting the role of histone deacetylation in epigenetic silencing of phenolic pathways (Nomura et al., 2021).

The molecular basis of HDAC mediated silencing can be interpreted as promoter-level hypoacetylation and reduced chromatin accessibility at phenylpropanoid pathway genes. When histone deacetylase activity is high, reduced acetylation of histone H3 and H4 limits access of transcription factors to promoters of genes such as PAL, CHS and methyltransferase genes (Sharma et al., 2019; Patrick et al., 2021; Su et al., 2021; Liu et al., 2023). In petunia flowers, the acetylation state of H3K9ac critically influences transcription within the shikimate derived volatile benzenoid/phenylpropanoid pathway. In this study, chemical repression of histone acetyl transferases (HAT) lowers H3K9ac, downregulates volatile pathway genes and reduces the accumulation of multiple phenolic compounds (Patrick et al., 2021). As inhibition of HAT will reduce the net acetylation status of histone, which has the same function of HDAC activity, these observations support the hypothesis that HDAC-mediated removal of H3K9ac represses shikimate pathway gene expression and thereby diminishing phenolic compound accumulation.

**Figure 5:**
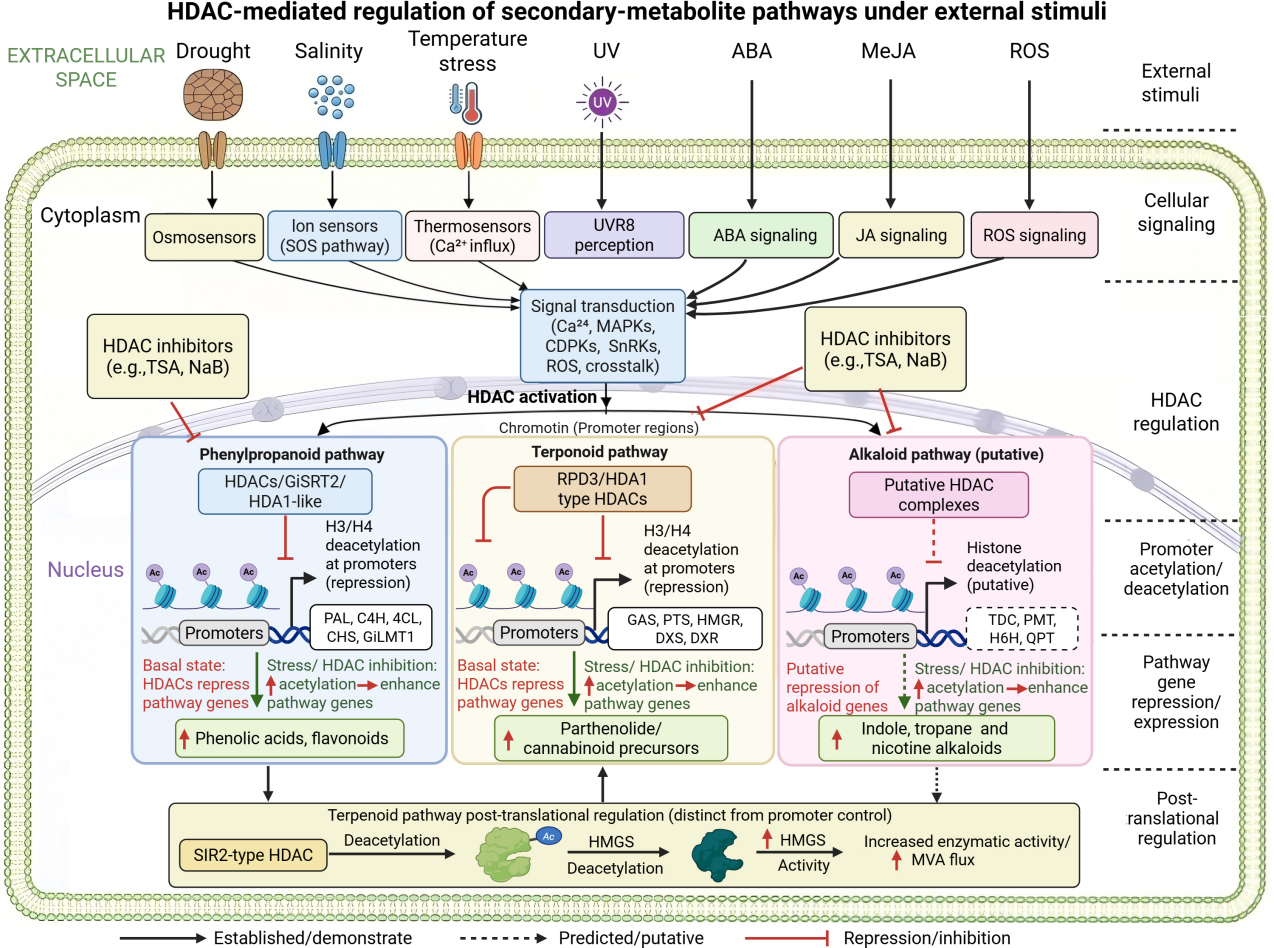
HDAC-mediated regulation of secondary-metabolite pathways under external stimuli. External stimuli activate intracellular signaling networks. At secondary-metabolite gene promoters, HDACs reduce H3/H4 acetylation and repress pathway genes under basal conditions, whereas stress or HDAC inhibitor treatment increases histone acetylation and de-represses genes involved in phenylpropanoid and terpenoid biosynthesis. SIR2-type HDACs also regulate HMGS through enzyme deacetylation, thereby increasing MVA pathway flux. HDAC regulation of alkaloid biosynthetic genes remains putative and requires further validation.

Under abiotic stresses, plants are known to up-regulate certain phenolic compounds to increase their resistance (Kumar et al., 2023). Meanwhile, it was also found that HDACs are important regulators in stress responses (Zeng et al., 2025). In the licorice study, MeJA and ABA treatment resulted in flavonoid accumulation in seedlings and this response was associated with increased H3K9, H3K14 and H3K27 acetylation together with reduced HDAC gene expression (Zeng et al., 2025). The study indicated that MeJA and ABA appear to suppress HDACs, thereby removing repression on phenylpropanoid pathway genes and allowing activation of flavonoid synthesis in response to stress. Similarly, under other abiotic stress conditions such as salinity, extreme temperature and cold, studies also revealed the functions of HDAC in stress responses (Hu et al., 2019; Jan et al., 2021). Mechanistically, under normal condition, HDACs repress and turn off phenolic compound biosynthetic pathway genes to maintain the level of required products. Under stress conditions, plants require higher level of phenolic compound and achieve that through increasing histone acetylation at the relevant genes by repressing HDACs. Once the stress is relieved, HDACs typically help restore the previous chromatin state, re-compacting chromatin and downregulating secondary metabolism genes to basal levels (Ibragić et al., 2025) (**Figure 5**). This reversible epigenetic switch ensures that phenolic defenses are organized rapidly during stress and conserved when conditions normalize.

Not all histone deacetylase effects are simple repression. In peanut (*Arachis hypogaea*) hairy root cultures, overexpression of an HDAC1 gene (*AhHDA1)* was associated with broad upregulation of flavonoid, isoflavonoid, and lignin-related genes (Su et al., 2021). The researchers hypothesize that this may be partly due to an increase in cellular acetyl-CoA levels generated by the heightened deacetylation activity. As an HDAC removes an acetyl group from histones, the freed acetyl moiety becomes acetyl-CoA, a key substrate for phenolic and terpenoid biosynthesis. Therefore, phenylpropanoid regulation by histone deacetylases should be discussed as a context-dependent process: some family members repress pathway genes directly, while others may promote secondary metabolism indirectly by changing carbon allocation, chromatin context, or upstream regulators (Su et al., 2021).

Generally, plant phenolic pathways are under multilayer controls: transcription factors and stress signals induce the activation of such pathway, after that, epigenetic mechanisms (histone deacetylation and DNA methylation) can dynamically repress or enhance phenolic compound accumulation. By affecting active HDACs, for instance, using HDAC inhibitors or editing repressor genes, is therefore a promising strategy to enhance the yield of valuable phenolics in medicinal plants (**Figure 5**; **Table 1**).

**Table 1:** HDAC regulation of secondary metabolites.

Plant species	HDAC or treatment	Metabolite effect	Mechanism/evidence
*Platycodon grandiflorus* (Ahn et al., 2023b; Ahn et al., 2023a)	NaB	↑ Phenolics/flavonoids	De-repression of phenylpropanoid genes
*Glycyrrhiza inflata* (Zeng et al., 2023; Zeng et al., 2025)	GiSRT2; nicotinamide	↑ licochalcone A Flavonoids	GiSRT2 suppresses GiLMT1 and related genes
Bamboo cells (Nomura et al., 2021)	SBHA, TSA	↑ cryptic phenolics	Activation of epigenetically silent pathways
Feverfew (*Tanacetum parthenium*) (Alimirzaee et al., 2024)	TSA	↑ parthenolide	Upregulation of GAS, PTS
Hemp (*Cannabis sativa*) (Yang et al., 2021)	TSA	Altered precursor levels	Altered expression of pathway genes
*Litsea cubeba* (Wu et al., 2021)	Sir2 overexpression	↑ terpenoids	HMGS enzyme deacetylation and activation
Fungi (*Cordyceps militaris*) (Kunhorm et al., 2022)	HDAC inhibitor	↑ cordycepin	Activation of silent biosynthetic clusters
*Petunia x hybrida* (Patrick et al., 2021)	H3K9ac balance	Changed benzenoid/phenylpropanoid volatiles	Chromatin acetylation controls pathway transcription
Peanut (*Arachis hypogaea*) (Su et al., 2021)	AhHDA1 overexpression	↑ flavonoid, isoflavonoid and lignin genes	Metabolic reallocation and chromatin remodeling
Rice *(Oryza sativa)* (Ullah et al., 2021; Guo et al., 2023)	HDA710; OsWR2-HDA704	ABA/salt/drought-responsive genes	Promoter-specific histone H4 deacetylation

### Terpenoid pathways and HDAC modulation

In terpenoid biosynthesis, HDACs regulate pathway output through both chromatin-dependent and protein-level mechanisms. In feverfew (*Tanacetum parthenium*), a medicinal herb producing the anti-migraine sesquiterpene lactone parthenolide, TSA treatment increased expression of MVA pathway enzymes germacrene A synthase (GAS), parthenolide synthase (PTS) and enhanced accumulation of parthenolide (Alimirzaee et al., 2024). In hemp (*Cannabis sativa*), 14 HDAC genes were identified across three families and expression profiling showed certain HDACs inversely correlate with cannabinoid synthesis in specific tissues (Yang et al., 2021). TSA treatment altered cannabinoid pathway gene expression and resulted in increased precursor accumulation (Yang et al., 2021). These findings support a transcriptional model in which histone deacetylation limits terpenoid pathway gene expression under basal conditions.

A second mechanism involves post-translational regulation of metabolic enzymes. It was found that HDACs can deacetylate not only histones but also a range of non-histone proteins (Jin et al., 2024). In *Litsea cubeba* (tested *via* overexpression in *N. benthamiana*), Sir2-type HDACs increased the type and amount of terpenoids by directly deacetylating and activating HMGS, a key enzyme of the MVA pathway (**Figure 5**) (Wu et al., 2021). This example illustrates how histone deacetylases can promote terpenoid biosynthesis even when other deacetylases repress pathway genes at the chromatin level. Within a single pathway, these two mechanisms may synergize if stress both de-represses biosynthetic genes and activates metabolic enzymes. They may also antagonize each other if one histone deacetylase represses transcription while another activates an enzyme. This explains why broad histone deacetylase inhibition and Sir2 overexpression can both increase terpenoid output in different systems (Wu et al., 2021; Alimirzaee et al., 2024). Future studies should therefore identify the specific histone deacetylase isoform and its molecular target (promoter, transcription factor or metabolic enzymes) to further illustrate their mechanisms.

### Alkaloid pathway and HDAC functional predictions

Compared to phenolics and terpenoids, direct evidence for HDAC mediated control of alkaloid biosynthesis in plants remains limited. Nevertheless, several lines of evidence support this possibility. Epigenetic control of secondary metabolism is often mediated through complex crosstalk between different chromatin modifications, where DNA methylation and histone acetylation often function as interconnected or opposing marks to establish transcriptional states. It was found DNA methylation plays important role in alkaloid biosynthesis, for instance, methylome and transcriptome analyses of *Catharanthus roseus* (the source of vinblastine/vincristine) revealed that DNA methylation patterns across the genome correlate with the expression of MIA pathway genes in a tissue-specific manner. In other words, epigenetic DNA marks help determine which tissues produce alkaloids by switching pathway genes on or off (Dugé de Bernonville et al., 2020). As a balancing strategy, HDAC may also contribute to such epigenetic regulations. Broad reviews of plant epigenetics and specialized metabolism highlight that histone acetylation dynamics and HDAC/HAT activities shape stress-responsive metabolic programs, providing a mechanistic basis for such involvement (Ibragić et al., 2025). As a result, while alkaloid-specific evidence is still lacking, insights from epigenetic regulation of plant specialized metabolism and from deacetylase control of related pathways strongly suggest an underexplored role for HDACs in alkaloid biosynthesis.

Evidence from fungi provides useful but indirect mechanistic support. In fungi, biosynthetic genes for a given secondary metabolite (including many alkaloids) are often physically clustered in the genome and co-regulated. HDACs generally repress these gene clusters by maintaining a condensed, hypoacetylated chromatin state (Xue et al., 2023). Inactivation of HDAC function either by chemical inhibitors or by genetic deletion frequently activates silent biosynthetic clusters, leading to increased production of secondary metabolites. For instance, *Cordyceps militaris* (an entomopathogenic fungus) showed a significant increase in the alkaloid-like compound cordycepin when treated with an HDAC inhibitor (Kunhorm et al., 2022). In *Penicillium* fungi, deletion of the class II HDAC gene hdaA in an endophytic *Penicillium chrysogenum* strain largely reduced the expression of a dominant alkaloid chrysogine (Ding et al., 2020). Interestingly, in the meanwhile, this mutant strain also showed increased expression of an indole alkaloid meleagrin, which was undetectable in the wild type. Meleagrin (and its precursor roquefortine C) are diketopiperazine alkaloids derived from tryptophan and proline, and their biosynthetic gene cluster was found to be upregulated in the HDAC knockout (Ding et al., 2020). This example illustrates that a single HDAC can act as a global modulator of secondary metabolism, repressing some pathways while indirectly enhancing others to activate. Although fungal alkaloids differ from plant ones, they share similar structures and biosynthetic origins. For instance, they share the initial step of tryptophan decarboxylation to tryptamine or a related indole intermediate. Moreover, fungi also produce alkaloids analogous to plant compounds in other classes. The *Penicillium chrysogenum* produced chrysogine belongs to a benzodiazepine derivative alkaloid, which is common in plants. The assembly of chrysogine *via* a nonribosomal peptide (NRPS) mechanism is similar to plant NRPS-derived alkaloids, such as cyclopeptide alkaloids in some plant families (Ding et al., 2020). As a result, plant HDAC family may perform some similar functions as the fungi HDACs, such as the repression roles of alkaloid pathway genes.

Based on current evidence, we propose a cautious model for plant alkaloids, where histone deacetylases may maintain basal repression of specific alkaloid genes in tissues or conditions where production is unnecessary. Under stress or elicitor treatment, MeJA-, ABA-, or reactive-oxygen-species-responsive transcription factors may reduce this repression, increasing histone acetylation and enabling expression of key alkaloid enzymes. This model remains hypothetical and should be tested by chromatin immunoprecipitation, histone-acetylation profiling, gene editing of candidate histone deacetylases, and combined transcriptome-metabolome analyses in alkaloid-rich medicinal plants such as *Catharanthus roseus*, opium poppy (*Papaver somniferum*), and *Hyoscyamus* species.

In summary, while direct evidence in medicinal plants is still limited, studies from microbial systems and the general principles of epigenetic gene regulation strongly suggest that HDACs are key modulators of a variety of secondary metabolite pathways. Based on the results, we proposed a model (**Figure 5**) in which HDACs act as key integrators of environmental signals, coordinating transcriptional regulation and protein function to control secondary metabolite biosynthesis.

## Discussion

### Environmental constraints and the need for mechanistic regulation

The motivation for studying histone deacetylases in medicinal plants is not only theoretical but also agronomic. Medicinal plant cultivation is often limited by habitat specificity, altitude, temperature, soil properties, water availability and biotic interactions (Schippmann et al., 2002; Hadjadj et al., 2022; Alum, 2024; Wang et al., 2025; Zhang et al., 2025). For example, some high value forest herbs show poor seed germination or depend on soil fungi under specific ecological conditions (Schippmann et al., 2002). While *Saussurea obvallata* is adapted to the cold, low oxygen environment of Qinghai-Tibet Plateau at high altitudes (3,200–4,700 m) (Zhang et al., 2025), and *Zygophyllum album*, native to Algerian deserts, is adapted to saline desert habitats. These constraints affect not only biomass production but also the stability of bioactive metabolites. Some plants, such as Danshen (*Salvia miltiorrhiza*), produce region-specific phytochemicals, with wild varieties often exhibiting greater potency than cultivated forms (Wang et al., 2025). Epigenetic regulation provides a mechanistic explanation for this instability, the environmental cues can reshape chromatin states at stress-response and metabolic genes, thereby changing pathway activity without altering genotype. Therefore, understanding HDAC-mediated regulation is important for improving plant adaptation, stabilizing compound quality and enhancing valuable secondary metabolite production under limited cultivation environment.

### Technological constraints and opportunities

Current cultivation and breeding technologies for medicinal plants remain less developed than those for major crops (Canter et al., 2005; Alamgir and Alamgir, 2017; Ramawat and Arora, 2021). The majority of medicinal plants in cultivation are still semi-wild types, only limited species have undergone substantial breeding and genetic improvement, leading to inconsistent yields and variable levels of active phytochemicals (Canter et al., 2005; Ramawat and Arora, 2021). Traditional breeding for traits such as seed viability, secondary metabolite yield, or resistance to abiotic stress is hampered by long generation cycles, low seed set and high heterozygosity. Moreover, molecular breeding tools like marker-assisted selection (MAS) are underutilized. While model crops have benefited from genome-wide association studies (GWAS) and next-generation sequencing, medicinal species rarely have even basic genetic maps (Canter et al., 2005; Alamgir and Alamgir, 2017). Insufficient propagation methods also significantly influence medicinal plant cultivation. For instance, American ginseng (*Panax quinquefolium*) requires stratification to break dormancy, the optimized environmental control can significantly improve germination (Li et al., 2000). Similarly, Indian snakeroot (*Rauvolfia serpentina*) exhibits variable germination (10–60%), with poor seed viability often linked to factors like hard seed coats or fungal infections (Alamgir and Alamgir, 2017). Vegetative propagation (*via* cuttings, suckers, rhizomes) is often used, but also with variable success. Although research showed hormone treatments like IBA or NAA can improve rooting (e.g., 78.5% success with stem-root junctions in *R. serpentina*) (Alamgir and Alamgir, 2017), these protocols are not standardized or widely used, particularly for small-scale farmers. In many cases, propagation still relies on wild-collected planting material, which can be genetically inconsistent or disease-prone (Ramawat and Arora, 2021). Furthermore, studies have shown that cultivated medicinal plants often exhibit differences in the content of bioactive compounds compared to their wild counterparts (Petropoulos et al., 2020), suggesting that current cultivation still requires more investigation to identify the underlying factors influencing the production of key secondary metabolites.

Histone deacetylase research can help address this gap by identifying regulatory genes that control broad transcriptional and metabolic programs. For species with limited breeding resources, chemical elicitation, transient inhibition, tissue-culture treatments, or targeted epigenetic editing may provide practical routes to enhance metabolite production before stable cultivars are available. However, global manipulation of histone deacetylases can also produce trade-offs. Since these enzymes regulate development, hormone signaling and stress responses, non-selective inhibition may impair growth or cause unwanted metabolic shifts. Therefore, future applications should prioritize isoform-specific targets, tissue-specific promoters, inducible systems and time-limited treatments. Such precision will be particularly important for medicinal plants in which the desired compound accumulates in specific organs, such as roots of Danshen (*Salvia miltiorrhiza*), stems of *Dendrobium officinale*, or aerial tissues of feverfew.

Another limitation is the lack of direct mechanistic validation in many medicinal plants. Most current studies report gene-family identification and expression patterns under stress but few of them combine chromatin immunoprecipitation, promoter acetylation assays, loss-of-function mutants, enzyme acetylation assays and metabolite profiling. Future work should move from correlation to causation by linking individual histone deacetylase isoforms with specific target genes, transcription factors, chromatin marks and metabolites. In addition, a comparative framework will be especially useful. In Danshen (*Salvia miltiorrhiza*), histone deacetylase genes can be studied in relation to phenolic acid and tanshinone biosynthesis; in *Dendrobium officinale*, they can be connected to stress-related polysaccharide and alkaloid-associated metabolism; in *Platycodon grandiflorus*, they can be examined in root metabolic responses to waterlogging; and in *Glycyrrhiza inflata*, GiSRT2 provides a concrete example of a histone deacetylase that negatively regulates flavonoid accumulation (Zhang et al., 2020; Ahn et al., 2023b; Zeng et al., 2023; Chen et al., 2024; Zeng et al., 2025). Comparing these systems will help determine whether histone deacetylase functions are conserved, species-specific, or pathway-specific.

### HDACs as strategic targets for secondary metabolite enhancement

The evidence summarized in this review supports histone deacetylases as strategic targets for improving secondary metabolite yield. In phenylpropanoid pathways, histone deacetylase inhibition or suppression often increases flavonoids, chalcones and phenolic acids by de-repressing pathway genes (Nomura et al., 2021; Patrick et al., 2021; Su et al., 2021; Ahn et al., 2023a; Zeng et al., 2023; Zeng et al., 2025). In terpenoid biosynthesis, histone deacetylases act through both transcriptional and post-translational mechanisms, with broad inhibition is shown to enhance gene expressions such as GAS and PTS, while Sir2-type proteins can activate HMGS through enzyme deacetylation (Wu et al., 2021; Alimirzaee et al., 2024). These two layers may be exploited together if the responsible isoforms and targets are clearly distinguished.

To further validate HDAC functions, several strategies can be considered. Chemical histone deacetylase inhibitors may be useful for cell cultures, hairy roots or short-term elicitation systems. Genetic approaches, including CRISPR-based knockouts, RNA interference, overexpression and dead Cas9-based epigenetic editing, may allow more precise control of individual histone deacetylase isoforms. For field applications, inducible or tissue-specific systems will be useful as they reduce the risk of growth inhibition. Eventually, the aim is to identify selective repressive HDAC isoforms that can be targeted while preserving, or even enhancing, metabolic enzyme activity and stress tolerance.

## Conclusion

HDACs are found to be key regulators of plant stress responses and secondary metabolism. Current evidence indicates that HDACs influence the biosynthesis of major metabolite classes, particularly phenolic compounds and terpenoids, through both transcriptional and post-translational mechanisms. Their roles in alkaloid biosynthesis remain largely hypothetical but are suggested by the broader principles of epigenetic control and evidence from methylation studies and fungal secondary metabolism. This review highlights the importance of HDACs as integrators of environmental signals and metabolic pathways. We propose that HDACs function as dynamic regulators that balance growth, stress adaptation and metabolite production through reversible chromatin modifications. Future studies should move beyond expression profiling to focus on isoform-specific functions, identify direct molecular targets, map histone acetylation at pathway genes, measure metabolite outcomes and validate these mechanisms under field conditions. Such studies will provide a foundation for using histone deacetylase mediated strategies to enhance stress resistance, yield and the stable production of valuable secondary metabolites in medicinal plants.

## Glossary

ABA: Abscisic acid
AhHDA1: *Arachis hypogaea* histone deacetylase 1
AN1: Anthocyanin 1 (MYB transcription factor)
AP2/ERF: APETALA2/Ethylene-Responsive Factor
bHLH: Basic helix–loop–helix (transcription factor family)
CAD: Cinnamyl alcohol dehydrogenase
CBF: C-repeat binding factor
C3H: *p*-Coumarate 3-hydroxylase
C4H: Cinnamate 4-hydroxylase
CCR: Cinnamoyl-CoA reductase
CHS: Chalcone synthase
CHI: Chalcone isomerase
CoA: Coenzyme A
CRISPR: Clustered Regularly Interspaced Short Palindromic Repeats
dCas9: Dead Cas9 (catalytically inactive Cas9)
DFR: Dihydroflavonol 4-reductase
DMAPP: Dimethylallyl pyrophosphate
DNA: Deoxyribonucleic acid
DoHDA/DoHDT: *Dendrobium officinale* histone deacetylase/HD2-type genes
DXP: 1-Deoxy-D-xylulose-5-phosphate
DXR: 1-Deoxy-D-xylulose-5-phosphate reductoisomerase
DXS: 1-Deoxy-D-xylulose-5-phosphate synthase
FPPS: Farnesyl pyrophosphate synthase
FtHDA6: *Fagopyrum tataricum* histone deacetylase 6
G3P: Glyceraldehyde-3-phosphate
GAS: Germacrene A synthase
GiLMT1: *Glycyrrhiza inflata* licochalcone A O-methyltransferase
GiSRT2: *Glycyrrhiza inflata* sirtuin-type histone deacetylase 2
GPPS: Geranyl pyrophosphate synthase
GWAS: Genome-wide association study
H3K9ac/H3K14ac/H3K27ac: Histone H3 lysine 9/14/27 acetylation
H6H: Hyoscyamine 6β-hydroxylase
HAT: Histone acetyltransferase
HD2: Plant-specific histone deacetylase family
HDAC: Histone deacetylase
HMG-CoA: 3-Hydroxy-3-methylglutaryl-CoA
HMGR: 3-Hydroxy-3-methylglutaryl-CoA reductase
HMGS: 3-Hydroxy-3-methylglutaryl-CoA synthase
IBA: Indole-3-butyric acid
IPP: Isopentenyl pyrophosphate
JA: Jasmonic acid
MeJA: Methyl jasmonate
MEP: Methylerythritol phosphate (pathway)
MAS: Marker-assisted selection
MIA: Monoterpene indole alkaloid
miRNA: MicroRNA
MVA: Mevalonate (pathway)
MYB: MYB transcription factor
NaB: Sodium butyrate
NAA: 1-Naphthaleneacetic acid
NAD⁺: Nicotinamide adenine dinucleotide
NRPS: Nonribosomal peptide synthetase
ODC: Ornithine decarboxylase
PAL: Phenylalanine ammonia-lyase
PMT: Putrescine N-methyltransferase
PTS: Parthenolide synthase
QPT: Quinolinate phosphoribosyltransferase
RNA: Ribonucleic acid
RPD3/HDA1: Reduced Potassium Dependency 3/Histone Deacetylase 1 family
SAHA: Suberoylanilide hydroxamic acid
SBHA: Suberoylbishydroxamic acid
SIR2: Silent Information Regulator 2
SM/SMs: Secondary metabolite(s)
SmHDAC: *Salvia miltiorrhiza* histone deacetylase
TDC: Tryptophan decarboxylase
TPS: Terpene synthase
TSA: Trichostatin A
UV: Ultraviolet
WRKY: WRKY transcription factor family.
